# Cognition malleability belief, emotion regulation and adolescent well-being: examining a mediation model among migrant youth

**DOI:** 10.1080/21642850.2020.1806717

**Published:** 2020-08-17

**Authors:** Shimin Zhu, Shiguang Ni, Kyra Hamilton

**Affiliations:** a Department of Applied Social Sciences, Hong Kong Polytechnic University, Kowloon, Hong Kong; b Department of Psychology, Tsinghua University, China; c School of Applied Psychology, Griffith University, Australia

**Keywords:** Well-being, emotion regulation, implicit beliefs, adolescent, migrant youth

## Abstract

**Objective:** The well-being of migrant youth is a major global public health concern. This developmental stage is fraught with many challenges, with migrant youth suffering additional challenges as a result of migration. One avenue to better understand the psychological mechanisms that underpin the well-being of migrant youth is examining how mindsets – or *implicit theories* about the malleability of human characteristics – affect well-being. The aim of the current study was to test a conceptual model in which cognition malleability belief on well-being would be mediated by emotion regulation styles in two samples of migrant youth using two different measures of well-being.

**Methods:** In Study 1, mainland China migrant youth (*N* = 735, Mean age = 13.89, SD = 1.23) completed a survey measuring demographics and cognition malleability belief, emotion regulation style (cognitive reappraisal, expressive suppression), and well-being (holistic well-being). In Study 2, Hong Kong migrant youth (*N *= 285, Mean age = 15.09, SD = 2.75) completed the same measures; however, well-being was assessed by the Life Satisfaction Scale. As different measures of the dependent variable (well-being) were used, two separate models were specified. Computations were performed with SPSS 22 and with the PROCESS macro.

**Results:** Both studies demonstrated support for the conceptual model. As predicted, cognition malleability belief was associated with cognitive reappraisal, expressive suppression, and well-being of migrant youth from mainland China and Hong Kong. Cognitive reappraisal was positively associated with well-being, while expressive suppression was not significantly associated with well-being. The association between cognition malleability belief and well-being was mediated by cognitive reappraisal.

**Conclusion:** Current findings provide avenues for future longitudinal and experimental research to test the efficacy of these mechanisms in changing beliefs about cognition malleability to promote the well-being of migrant youth.

## Introduction

The well-being of migrant youth is a major public health concern globally (Harttgen & Klasen, 2009), especially given the increasing number of migrant youth worldwide, with global reports estimating more than thirty million migrant youth aged 15–24 years (ISSOP Migration Working Group, 2018; UN Department of Economic and Social Affairs, 2016). The issue is particularly extensive in mainland China and Hong Kong. In mainland China, it is estimated that up to thirty-five million rural children aged between 1 and 17 years have migrated to urban areas, often as a result of extensive urbanization (Chan & Ren, 2018; Wei, Wang, Chen, & Wang, 2014). In Hong Kong, records show that 14,042 children are migrant children from mainland China (Constitutional and Mainland Affairs Bureau, 2019), often due to cross-border marriage.

Although migration may provide new and positive opportunities, hence the term the ‘healthy migrant’ effect (Kearns, Whitley, Egan, Tabbner, & Tannahill, 2017), migrant youth are often confronted with challenges such as poorer living standards, lower education opportunities, and social exclusion and discrimination (Li, Stanton, Fang, & Lin, 2006; Mao & Zhao, 2012; United Nations, 2013; Wei et al., 2014), which can have detrimental consequences on adolescent well-being. In addition, this developmental stage is fraught with many other challenges such as body changes, identity issues, and greater schooling and family demands, which may also cause stress and higher demands on emotion regulation (Dick & Ferguson, 2015). It is therefore not surprising that migrant youth report higher stress levels and lower self-worth (Harttgen & Klasen, 2009; Mao & Zhao, 2012) and present with greater mental health problems and lower well-being than local counterparts (see a review, Sun, Chen, & Chan, 2016). This, in turn, can lead to a range of maladaptive behavioral outcomes such as engagement in health-risk behaviors (e.g. physical inactivity, alcohol misuse, poor sleep) (Mood, Jonsson, & Låftman, 2016), increased mental health issues (e.g. depression, anxiety), and poor coping strategies (e.g. avoidance coping) (Schneiderman, Ironson, & Siegel, 2005). Given these challenges and potential for poor outcomes, it is important that further research is conducted to better understand the psychological mechanisms that underpin the well-being of migrant youth.

One potential avenue of investigation is examining how malleability beliefs affect adolescent well-being. Malleability beliefs refer to individuals’ beliefs about the malleability of human characteristics; i.e. human attributes are malleable and, therefore, can be improved or developed (Kneeland & Dovidio, 2019), also referred to as implicit theories or growth mindsets (Dweck, 2006). Research has shown more positive outcomes of holding malleability beliefs over non-malleability beliefs (i.e. beliefs that human attributes are fixed or unchangeable), including greater academic achievement (Dweck, 2006; Kneeland, Dovidio, Joormann, & Clark, 2016a), higher self-regulation (see a review, Burnette, O'Boyle, VanEpps, Pollack, & Finkel, 2013), and better judgement and reaction (Dweck, Chiu, & Hong, 1995). Also, a meta-analysis found non-malleability beliefs to be associated with more internalizing problems including anxiety and depression, and more externalizing youth problems including conduct and behavioral difficulties (see a review, Schleider, Abel, & Weisz, 2015), better emotion regulation (Schroder, Dawood, Yalch, Donnellan, & Moser, 2015), and psychological well-being (De Castella et al., 2013; Keech, Hagger, O’Callaghan, & Hamilton, 2018). Thus, malleability beliefs not only have the potential to improve individuals’ well-being but may also exert an influence on individuals’ emotion styles, such as emotion regulation (Kneeland et al., 2016a; Schleider et al., 2015; Schroder et al., 2015).

Specifically, emotion regulation may be an important mediator of the association between malleability belief and migrant youth well-being. Kneeland and colleagues (2016a) developed a model to conceptualize the process of how emotion malleability beliefs might influence emotionality, emotion regulation, and psychopathology, proposing that malleability beliefs are associated with more active emotion regulation styles, such as cognition reappraisal (i.e. reframing the way one thinks about a particular event), and less response-focused regulatory styles, such as expressive suppression (i.e. attempting to hide any sign of outward emotional expression). The use of expressive suppression, in particular, is thought to produce greater cognitive depletion or reduced mental energy to engage in self-regulation (Gross, 2002). Empirical research has found support for the model. For example, in a sample of university students it was demonstrated that those with malleable emotion beliefs engaged more in cognitive reappraisal and had greater positive affect and less anxiety during a task of giving a speech, but no significant effect for the use of expressive suppression was found (Kneeland, Holen-Hoeksema, Dovidio, & Gruber, 2016b). Another study found that emotion regulation played a significant role in linking emotion malleability beliefs to depression. University students’ beliefs that emotions were more malleable at the beginning of the semester predicted less depression at the end of the semester through greater use of cognitive reappraisal (Kneeland & Dovidio, 2019).

### The current study

The aim of the current study was to examine a conceptual mediation model of cognition malleability belief and well-being among migrant youth in mainland China and Hong Kong. The study addresses three gaps in extant literature. First, current literature has tended to focus on the impact of malleability beliefs on mental health problems rather than on well-being. Well-being has been acknowledged to be an important indicator of mental health, where an individual realizes his or her ability to cope with the normal stresses of life (Slade, 2010; World Health Organization, 2014). The current study filled this knowledge gap by examining the association between cognition malleability belief and well-being. Second, little is currently known about how malleability beliefs are linked to the well-being of migrant youth. Addressing this gap, the current study collected data from migrant youth from two regions – mainland China and Hong Kong Special Administrative Region – to provide evidence of the association between cognition malleability beliefs and well-being among migrant youth. This formative evidence may help to guide future prevention and early intervention efforts aimed at promoting the well-being of migrant youth. Third, although previous research has demonstrated a link between emotion malleability belief and well-being (De Castella et al., 2013), little is known about how cognition malleability belief may be related to well-being. People’s malleability beliefs, that may predispose them toward emotion regulation strategies, have important consequences for psychological health (De Castella et al., 2013). In particular, cognition malleability belief may be an important factor that explains individual differences in the use of adaptive emotion regulation like cognitive reappraisal. The current study, therefore, focused on the association among cognition malleability belief and emotion regulation and well-being.

In sum, although there is no direct literature testing the effect of malleability beliefs on the well-being of migrant youth, we hypothesized, based on the theorizing above, that malleability beliefs about cognition would be associated with higher subjective well-being of migrant youth, and that this association would be mediated by emotion regulation style. We measured both cognitive reappraisal and expressive suppression as two styles of emotion regulation. To provide some evidence for consistency of model effects and to enhance generalizability of findings to different migrant youth populations (Check & Schutt, 2012), we conducted the study in two samples of migrant youth (mainland China and Hong Kong), and used different measures of subjective well-being. Study 1 used the Holistic Well-being Scale (Chan et al., 2014) and Study 2 used the Satisfaction with Life Scale (Diener, Emmons, Larsen, & Griffin, 1985) to measure subjective well-being in migrant youth in mainland China and Hong Kong, respectively. As different measures of the dependent variable were used, two separate models were specified.

## Methods

### Design, participants, and procedures

Ethical approval was obtained from the University's Institutional review board of the second author . A cross-sectional design was used, with two surveys being administered to two samples of migrant youth in Shenzhen city and Hong Kong. Eligibility criteria included being a migrant youth defined as someone who had migrated to cities from their hometown and had lived in the urban city they had migrated to for at least six months. Both parent and child consent were required for participation. The survey took about 20–25 min to complete, and students completed the survey in class time under the instruction of a trained research assistant. All students received a notebook and a pencil as a thank-you gift for their time and effort.

In study 1, the sample comprised 735 migrant youth (*M_age_* = 13.91 years, *SD* = 1.26, range = 11–17 years; 52% male; 26% grade seven, 36% grade eight, and 37% grade nine) from three non-government funded secondary schools that specifically accommodate migrant students in Shenzhen, Guangdong Province, China. Ninety-six per cent of the sample identified as Han and 6% as minor ethnic groups, with 13.4% of the sample belonging to only-child families.

In Study 2, the sample comprised 285 migrant youth (*M_age_* = 15.09 years, *SD* = 2.75, range = 10–19 years; 55% male; 34% grade seven, 43% grade eight, and 23% grade nine) from eight secondary schools in Hong Kong, China. All were migrants from mainland China, 55% from Guangdong province and 45% from other mainland provinces, and 36.6% of the sample were from only-child families.

### Measures

*Cognition malleability belief* was measured in Study 1 and 2 by four items using a Chinese version based on the Implicit Theories of Thoughts Questionnaire (Schleider & Weisz, 2016; Zhu, Zhuang, & Cheung, 2020), scored *strongly disagree* (1) to *strongly agree* (7). A sample item is ‘When you don’t like the thoughts you have, you can change them.’ A higher mean score reflects a greater belief that cognition is changeable and malleable. The scale was translated and back-translated to ensure language accuracy and pilot tested to ensure understandability. Cronbach’s alpha for Study 1 was .67. Cronbach’s for Study 2 was .85.

*Emotion regulation* was measured in Study 1 and 2 using the Emotion Regulation Questionnaire (ERQ) (Gross & John, 2003; Wang, Liu, Li, & Du, 2007). The ERQ measures two dimensions of emotion regulation, namely cognitive reappraisal and expressive suppression, each assessed with seven items and scored *strongly disagree* (1) to *strongly agree* (7). A sample item for cognitive reappraisal is ‘I control my emotions by changing the way I think about the situation I’m in.’ and a sample item for expressive suppression is ‘I control my emotions by not expressing them.’ A higher mean score reflects a greater agreement to adopt that particular emotion regulation style. Cronbach’s for Study 1was .79 (cognitive reappraisal) and .72 (expressive suppression). Cronbach’s for Study 2 was .88 (cognitive reappraisal) and .79 (expressive suppression).

*Well-being* was measured in Study 1 by using the four-item Holistic Well-being Scale developed for Chinese adolescents (C. H. Y. Chan et al., 2014), scored *strongly disagree* (1) to *strongly agree* (9). A sample item is ‘I have a healthy body.’ A higher mean score reflects a greater level of perceived holistic well-being. Cronbach alpha for was .85. In Study 2, well-being was measured using the Satisfaction with Life Scale (SWLS) (Diener et al., 1985), scored *strongly disagree* (1) to *strongly agree* (7). A sample item is ‘The conditions of my life are excellent.’ A higher mean score reflects a greater level of perceived satisfaction with life. Cronbach alpha was .77.

### Data analysis

Computations were performed with SPSS 22 and with the PROCESS macro (Hayes, 2013). First, descriptive analyses and bi-variate correlations among main variables were examined. Second, to explore the multiple mediation hypothesis, a manifest variable model was specified in which cognitive reappraisal and expressive suppression as putative mediators were regressed on cognition malleability belief; whereas well-being (measured using the Holistic Well-being scale) was regressed on cognition malleability belief and the two mediators (i.e. cognitive reappraisal and expressive suppression), controlling for age and sex. A mediation model (model 4) using Nonparametric Bootstrap of Conditional process analysis in the PROCESS macro was executed, while confidence intervals (95%) generated by bootstrapping with 5,000 re-samples (Hayes & Rockwood, 2017). If the upper and lower bounds of interval do not include zero, then the hypothesis is supported (Hayes, 2013). A hot deck imputation procedure was applied to replace missing values, in which imputes a missing value with the value from a randomly selected case similarly to the missing case (Hawthorne & Elliott, 2005).

## Results

### Study 1

Means, standard deviations, and intercorrelations are shown in Table 1. As displayed in Table 1, cognition malleability belief was positively associated with both cognitive reappraisal (*r* = .38, *p* < .01) and expressive suppression (*r* = .17, *p* < .01). Cognition malleability belief and cognitive reappraisal, but not expressive suppression, were significantly and positively correlated with well-being.

**Table 1.** Correlations and Descriptive Statistics for the Main Variables of Study 1.

	Mean or %	*SD*	Sex	Age	Cognition malleability belief	Cognitive reappraisal	Expressive suppression	Well-being
Sex (male%)	52%	–	–					
Age(in years)	13.89	1.23	−.07	–				
Cognition malleability belief	2.94	1.04	.03	.01	–			
Cognitive reappraisal	4.74	1.04	.08*	.01	.38**	–		
Expressive suppression	4.21	1.05	-.08*	.07	.17**	.49**	–	
Well-being	6.18	2.24	.02	.00	.16**	.15**	−.03	–

Note: *p < .05, **p < .01;

Male = 1, Female = 2. The scores of Cognition malleability belief scale, cognitive reappraisal, and expressive suppression range from 1 to 7 with high scores respectively mean stronger beliefs of cognition malleability, more use of cognitive reappraisal and more use of expressive suppression as emotion regulation. Well-being was measured by Holistic Well-being Scale, ranged from 1 to 9. Higher score means more self-perceived well-being.

**Figure 1.**
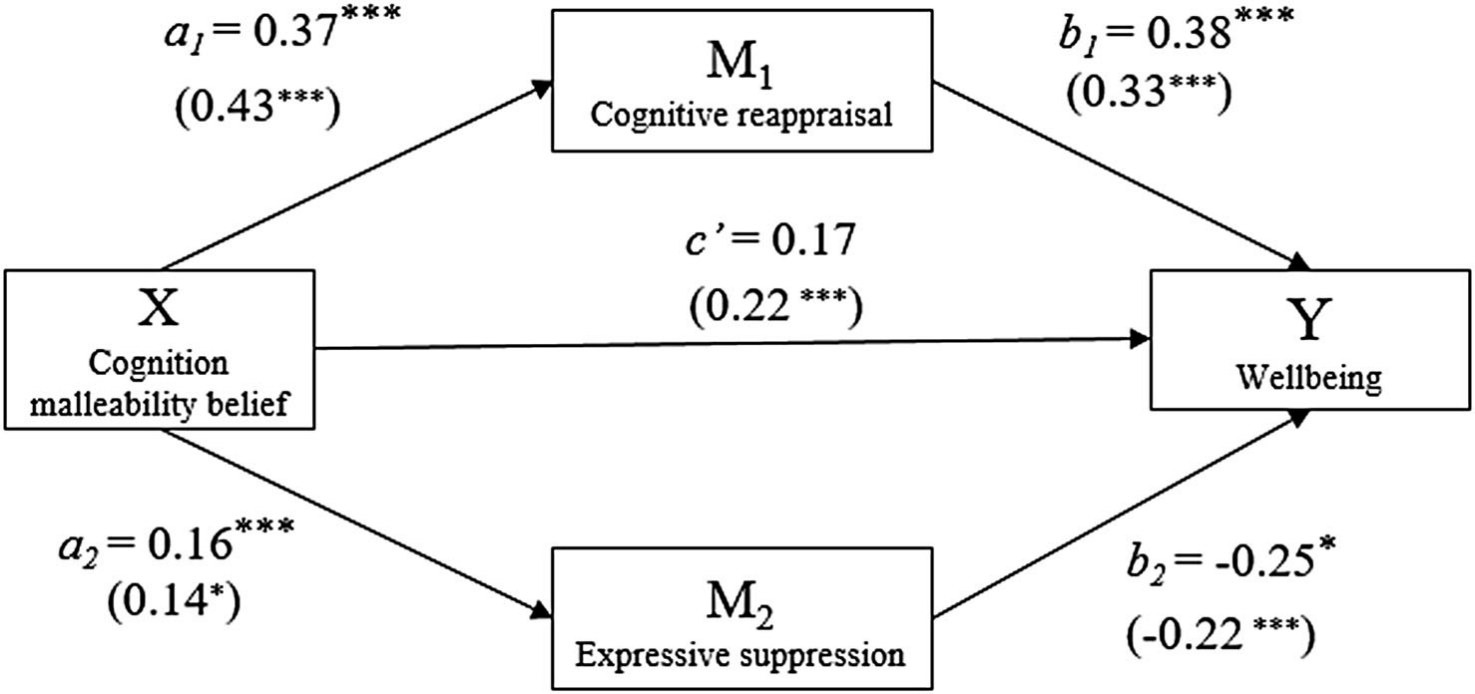
Model coefficient results of the bootstrapping analysis. Note. Coefficients are unstandardized. *** *p* < 0.001, * *p* < 0.05. Statistics in parentheses are results from Study 2.

Testing the multiple mediation hypothesis (see Table 2 and Figure 1), the following unstandardized parameters were estimated. The effect of cognition malleability belief on cognitive reappraisal was *b_ _*= .37, CI 95% (.29, .44); the effect of cognition malleability belief on expressive suppression was *b_ _*= .16, CI 95% (.08, .24); the effect of cognitive reappraisal on well-being was *b_ _*= .38, CI 95% (.17, .59); and the effect of expressive suppression on well-being was *b_ _*= −.25, CI 95% (−.45, −.06). The total indirect effect of cognition malleability belief on well-being via the two mediators was *b* = .10, Boot CI 95% (.02, .19): the indirect effect from cognition reappraisal (bootstrap mean = .14, 95% CI = .05 to .24) and the indirect effect from expressive suppression (bootstrap mean = −0.04, 95% CI = −.10 to −.01). Of the well-being variance, 10% was accounted for by this set of predictors. Controlling for age and sex did not change the results.

**Table 2.** Regression coefficients, standard errors, and model summary information for the conceptual model of study 1.

Predictors	Consequent													
	M_1_ (Cognitive reappraisal)					M_2_ (Expressive suppression)					Y (Well-being^a^)			
		Coeff.	*SE*	*P*			Coeff.	*SE*	*p*			Coeff.	*SE*	*p*
Constant	*i_M1_*	3.43	.50	<.001		*i_M_*_2_	3.32	.54	<.001		*i_Y_*	5.72	1.20	<.001
X	*a_1_*	.37	.04	<.001		*a*_2_	.16	.04	<.001		*c’*	.17	.09	<.05
M_1_											*b_1_*	.38	.11	<.001
M_2_											*b_2_*	−.25	.10	<.05
Sex		.17	.08	> .05			−.16	.09	> .05			−.13	.19	> .05
Age		-.002	.03	> .05			.05	.04	> .05			−.04	.08	> .05
		*R*^2 ^= .14					*R*^2 ^= .03					*R*^2 ^= .04		
		*F*_(3,567)_ = 30.74, *p* < .001					*F*_(3,567)_ = 6.36, *p* < .001					*F*_(5,565)_ = 4.75, *p* < .001		

Note: X = Cognition malleability belief, M1 = Cognitive reappraisal, M2 = Expressive suppression.

^a^Wellbeing was measured using the Holistic Well-being scale, scored on a 9-point scale.

The results of Study 1 supported the mediation hypothesis; cognition malleability belief was associated with well-being and this association was mediated by emotion regulation. Specifically, the model showed that for migrant youth in mainland China, believing cognition is changeable and malleable was associated with higher levels of well-being when a cognition reappraisal style was adopted. Also, it is found that the more the participants believe cognition can change, the more likely they used expressive suppression as emotion regulation. This may relate to the motivation of emotion regulation, but cognitive reappraisal is positively associated with well-being, which is similar to literature (Kneeland & Dovidio, 2019; Kneeland et al., 2016b).

### Study 2

Means, standard deviations, and correlations are shown in Table 3. As displayed in Table 3, cognition malleability belief was positively associated with cognitive reappraisal (*r* = .49, *p* < .01) and expressive suppression (*r* = .17, *p* < .01). Cognition malleability belief and cognitive reappraisal, but not expressive suppression, were significantly and positively correlated with well-being.

**Table 3.** Correlations and Descriptive Statistics for the Main Variables of Study 2.

	Mean or %	*SD*	Sex	Age	Cognition malleability belief	Cognitive reappraisal	Expressive suppression	Well-being
Sex ^a^ (male%)	55%	–	–					
Age (in years)	15.09	2.75	−.08	–				
Cognition malleability belief	4.90	1.03	.002	−.16**	–			
Cognitive reappraisal	4.57	.94	.04	−.16**	.49**	–		
Expressive suppression	4.21	.92	−.11	−.06	.17**	.48**	–	
Well-being	4.13	.99	.05	−.21**	.39**	.36**	.01	–

Note: **p < .01.

^a^Male = 1, Female = 2. The scores of Cognition malleability belief scale, cognitive reappraisal, and expressive suppression range from 1 to 7 with high scores respectively mean stronger beliefs of cognition malleability, more use of cognitive reappraisal and more use of expressive suppression as emotion regulation. Well-being was measured by Satisfaction with Life Scale (SWLS), ranged from 1 to 7. Higher score means more self-perceived satisfactory with life.

Testing the mediation hypothesis (see Table 4 and Figure 1), the following unstandardized parameters were estimated. The effect of cognition malleability belief on cognitive reappraisal was *b *= .43, CI 95% (.33, .53); the effect of cognition malleability belief on expressive suppression was *b* = .14, CI 95% (.03, .25); the effect of cognitive reappraisal on well-being was *b* = .33, CI 95% (.19, .48); and the effect of expressive suppression on well-being was *b* = -.22, CI 95% (−.35, −.09). The total indirect effect of cognition malleability belief on well-being via the two mediators was *b *= .11, Boot CI 95% (.05, .19): the indirect effect from cognition reappraisal (bootstrap mean = .14, 95% CI = .08 to .22) and the indirect effect from expressive suppression (bootstrap mean = −.03, 95% CI = −.08 to −.00). Of the well-being variance, 11% was accounted for by this set of predictors. Controlling for age and sex did not change the results.

**Table 4.** Regression coefficients, standard errors, and model summary information for the conceptual model of study 2.

Antecedent	Consequent											
	M_1_ (Cognitive reappraisal)				M_2_ (Expressive suppression)				Y (Satisfaction in life)^a^			
		Coeff.	*SE*	*p*		Coeff.	*SE*	*p*		Coeff.	*SE*	*p*
Constant	*i_M1_*	2.93	.44	<.001	*i_M2_*	4.20	.48	<.001	*i_Y_*	3.07	.54	<.001
X	*a_1_*	.43	.05	<.001	*a_2_*	.14	.06	<.05	*c’*	.22	.06	<.001
M_1_									*b_1_*	.33	.07	<.001
M_2_									*b_2_*	−.22	.06	<.001
Sex		.04	.10	>.05		−.23	.11	<.05		.01	.11	> .05
Age		−.04	.02	>.05		−.02	.02	> .05		−.04	.02	> .05
		*R^2^*^ ^= 0.23				*R^2^*^ ^= 0.04				*R^2^*^ ^= 0.22		
		*F*(3,261) = 25.50, *p* < 0.001				*F*(3,261) = 3.89, *p* < 0.05				*F*(5,259) = 14.21 *p* < 0.001		

Notes: X = Cognition malleability belief, M_1_ = Cognitive reappraisal, M_2_ = Expressive suppression.

^a^Well-being was measured using the Satisfaction with Life Scale, scored on a 7-point scale.

The results of Study 2 provided further support for the mediation hypothesis. Similar to Study 1, the model in Study 2 showed that for migrant youth from mainland China in Hong Kong holding the belief that cognition is changeable and malleable was associated with higher levels of well-being and this association is positively mediated by cognition reappraisal.

## Discussion

The well-being of migrant youth is an important area of investigation, yet currently is under research. This developmental stage alone is fraught with many challenges and migrant youth are faced with additional hurdles as a result of the demands from migration. As malleability beliefs have been shown to play an important role in adjustment, the aim of the current study was to test a mediation model of cognition malleability belief in which the said beliefs of migrant youth in both mainland China (Study 1) and Hong Kong (Study 2) affect individual well-being and that the process is mediated by emotion regulation (cognitive reappraisal and expressive suppression). Results of both samples suggest that cognition malleability belief is a distinct and important variable in determining the perceived well-being of migrant youth and that this relationship is mediated by emotion regulation, mainly cognitive reappraisal. The two successive studies, using different sample and different measures of subjective well-being, provide more evidence to support the generalizability of the conceptual model.

A positive association between cognition malleability belief and expressive suppression was also found. The results confirmed that the more one believes cognition is malleable, the more likely they are to adopt cognitive reappraisal and expressive suppression as emotion regulation strategies. There may be two reasons for this association. On the one hand, cognitive reappraisal and expressive suppression are two different domains of emotion regulation. Individuals may vary in their motivation to emotion regulation. People, who have high motivation to regulate their emotion, may engage their effort in reappraising their thoughts or supressing expression of emotion (Compas et al., 2017). If one holds cognition malleability beliefs, they may choose cognitive reappraisal as an emotion coping strategy, which is more congruent to their belief (Kneeland et al., 2016a) and, therefore, may try to control their emotions and not to express them. It is also possible that after engaging in cognitive appraisal, some young people may find their emotions less distressing and, hence, feel less urge to express them. On the other hand, migrant youth may be more vulnerable to having a strong social network and/or obtaining social support and, therefore, find it difficult to find someone with whom they can express their emotions (Zeman, Cassano, Perry-Parrish, & Stegall, 2006). Thus, it may be that migrant youth who held cognition malleability beliefs and make cognitive appraisals use less emotional expression as an emotion regulation strategy.

The current study, to the authors’ knowledge, is the first to investigate the associations of cognition malleability belief, emotion regulation, and migrant youth well-being. Previous literature has provided evidence for an association between implicit theories and psychopathology and mental health (Kneeland et al., 2016a; Schleider et al., 2015). This study extends previous research by providing empirical evidence for an association between implicit theories and well-being, specifically that holding a belief that cognition is malleable is associated with migrant youth subjective well-being. Current findings, therefore, provide further support for the theorizing that implicit theory is related to mental states. Moreover, current results indicate that the relationship between cognition malleability belief and migrant youth well-being is mediated by emotion regulation. Specifically, holding the belief that cognition is changeable and malleable is associated with greater levels of well-being if adopting a cognition reappraisal style and lower well-being if adopting an expressive suppression style. These findings have important implications for both theory and practice.

From a theoretical perspective, this study presents a new direction of the impact of implicit theory on mental health issues and well-being (De Castella et al., 2013; Kneeland et al., 2016a; Schleider et al., 2015). Echoing the evidence of the negative effect of non-malleability belief on mental health (De Castella et al., 2013; Schleider et al., 2015; Schroder et al., 2015), the findings of the current study support that cognition malleability beliefs are positively associated with well-being. Further, this study extends the focus from emotion malleability belief to cognition malleability belief; it integrates cognitive malleability belief, emotion regulation, and well-being to further our understanding of how thoughts influence one’s subjective well-being. In addition, current findings provide support for the theorizing underpinning Kneeland et al.’s (2016) mediation model of emotion regulation. Individuals who believe cognition can change are more likely to adjust their thoughts. The cognitive malleability belief may not only increase one’s sense of control over his or her thoughts and encourage efforts to try to reappraise those thoughts, it may also increase optimism about the effectiveness of the cognitive reappraisal and belief that effort of changing one’s thoughts would not be in vain (Kneeland et al., 2016a). Finally, current results provide formative evidence that suggests cognition reappraisal and expressive suppression may play different roles in mediating the impacts of cognition malleability belief.

From a practical perspective, current findings may inform the development of future interventions aimed at improving migrant youth well-being. First, the present study indicated that migrant youth might benefit from psychoeducation on the malleability of their cognition. Building the mindset of believing that one’s cognition can change would be helpful for adolescents to become more motivated for self-adjustment and self-regulation when encountering struggles (Compas et al., 2017; De Castella et al., 2014; Keech, Hagger, & Hamilton, 2019; Kneeland et al., 2016a). Second, as believing that cognition malleability belief can play an important role in cognitive reappraisal, school counseling programs might benefit from assessing students’ belief in this context to deliver more effective counseling strategies. For example, if a youth is assessed as having a fixed mindset, it might be useful to first work on developing the belief that human attributes are malleable and therefore can be improved or developed before tackling other issues. Third, this study demonstrated that emotion regulation leverages the impact of mindset on well-being. Although cognition malleability belief plays a role in well-being, this impact is mediated by emotion regulation. Current findings indicated a positive association between cognitive malleability belief and wellbeing was mediated by cognitive reappraisal. This suggests that individuals who believe cognition is changeable may be more likely to reappraise their thoughts thereby maintaining wellbeing status. Thus, current findings may provide insights into the effective use of cognitive behavioral therapy for improving individuals’ well-being. For example, holding cognitive malleability beliefs may lead to more effective therapy outcomes and, thus, may be a necessary parameter to determine whether using cognitive behavioral therapy is applicable given the situation. Future research is needed to further explore this idea.

Some conceptual and methodological limitations of the current study need to be mentioned. Both studies use concurrent designs, which is problematic for tests of mediation models (Cole & Maxwell, 2003). Also, the cross-sectional nature of the studies does not allow inferences of causal effects. Future research should test the model using longitudinal designs to confirm the proposed mediation pathways. The measures used in the current study relied exclusively on self-reports which may be subject to acquiescence response bias. In addition, although we used two different population groups to provide some evidence of consistency of effects and, thus, providing stronger evidence in support of the findings, we acknowledge that due to the use of different measures of the dependent variable (well-being), the model could not be specified as a multi-group structural equation model and measurement invariance testing could not be conducted. Further, the sample comprised migrant youth from mainland China and Hong Kong, thus limiting the generalizability of data across other migrant youth groups. Future research should continue to explore whether disparities, as well as similarities, exist in other populations. In addition, the focus of this study was on well-being. Cognition malleability belief may affect other mental states via pathways other than through emotion regulation (Kneeland et al., 2016a). These areas deserve future attention.

In conclusion, the well-being of migrant youth is a concern. Current findings provide further evidence that mindsets matter on health and well-being; however, the influence of this effect may be through one’s emotion regulation. This study provides avenues for future research to develop interventions for testing the efficacy of these mechanisms in promoting beliefs about cognitive malleability beliefs to ensure the well-being of migrant youth.
